# Two-Hour Lactate Clearance Predicts Negative Outcome in Patients with Cardiorespiratory Insufficiency

**DOI:** 10.1155/2010/917053

**Published:** 2010-06-28

**Authors:** Sean Scott, Vittorio Antonaglia, Giovanna Guiotto, Fiorella Paladino, Fernando Schiraldi

**Affiliations:** ^1^Emergency Department, Royal North Shore Hospital, Sydney, Australia; ^2^Department of Anaesthesia and Intensive Care, University of Trieste, Trieste 34123, Italy; ^3^Department of Emergency Medicine and Critical Care, San Paolo Hospital, Naples, Italy

## Abstract

*Objective*. To evaluate 2-hour lactate clearance as a prognostic marker in acute cardiorespiratory insufficiency. *Design*. Prospective observational study. *Setting*. Emergency Department (ED) and 16-bed medical High Dependency Unit (HDU). *Methods and Main Results*. 95 consecutive admissions from the ED for acute cardiorespiratory insufficiency were prospectively enrolled. Arterial lactate concentration was assessed at ED arrival and 1, 2, 6, and 24 hours later. The predictive value of 2-hour lactate clearance was evaluated for negative outcomes defined as hospital mortality or need for endotracheal intubation versus positive outcomes defined as discharge or transfer to a general medical ward. Logistic regression and ROC curves found 2-hour lactate clearance >15% was a strong predictor of negative outcome (*P* < .0001) with a sensitivity of 86% (95%CI = 67%–95%) and a specificity of 91% (95%CI = 82%–96%), Positive predictive value was 80% (95%CI = 61%–92%), and negative predictive value was 92% (95%CI = 84%–98%). *Conclusions*. Systematic monitoring of lactate clearance at 2 hours can be used in to identify patients at high risk of negative outcome and perhaps to tailor more aggressive therapy. Equally important is that a 2-hour lactate clearance >15% is highly predictive of positive outcome and may reassure clinicians that the therapeutic approach is appropriate.

## 1. Introduction

Acute cardiorespiratory failure from any cause creates an imbalance between energy supply, demand, and consumption. A high lactate concentration associated with a low blood pH is useful to depict the gravity of such a mismatch [1]. Lactate overproduction can be promptly removed from blood if renal and hepatic perfusion and function are near normal [2, 3]. A huge bulk of research has been focused on lactate production/removal in critically ill patients. Some evidence suggests that persistent elevation in lactate is associated with high mortality rate [4–6]. It is generally agreed that the longer lactate levels remain high, the worse the prognosis [7, 8]. However, a question remains regarding the timing needed to guide the therapy based on evaluation of lactate clearance [9]. 

Following the concept of “Early-Goal-Directed-Therapy” (EGDT) [10], one should be satisfied if the therapeutic approach determines a lactate clearance greater than 20% within 6 hours of treatment (LACT-6h-clearance). Indeed, this metabolic goal is acceptable when the lactate over-production reflects a hypermetabolic state, as in septic patients. However, LACT-6h-clearance is less useful in assessing the response to treatment when an increase in lactate occurs rapidly, as in patients with acute cardiorespiratory failure. 

We hypothesized that, in these patients, an earlier lactate clearance should be useful to assess the metabolic response to therapy and to identify patients able to reverse the metabolic derangement. A secondary hypothesis was that failure to rapidly resolve the oxygen debt, indicated by poor lactate clearance, would be correlated with negative outcome. For this purpose a prospective series of patients consecutively admitted to the Emergency Department with acute cardiorespiratory failure underwent a standardized therapeutic approach and the metabolic response was assessed by lactate clearance after 2 hours of treatment (LACT-2h-clearance). The first aim of the study was to evaluate the utility of short-term lactate clearance in acute cardiorespiratory failure patients and the second was to assess its value in predicting patient outcome.

## 2. Materials and Methods

### 2.1. Inclusion Criteria

Acute decompensated cardiac failure and/or “acute-on-chronic” respiratory failure. Acute cardiac failure was defined as a condition with decreased tissue perfusion other than simple hypovolemia in which volume and/or drugs that improve cardiac performance and/or restore abnormal vascular tone could be prescribed. Respiratory failure was defined as oxygen saturation of less than 90% despite supplementary oxygen with or without hypercapnia. 

These entities were defined clinically in the emergency department with at least 4 out of 6 of the following parameters:

 Respiratory rate >25
 Heart rate >130
 Systolic blood pressure <90
 SpO_2_<90% at FiO_2_ of 0.30
 Bibasal crepitations on auscultation
 Arterial blood lactate >2 mmol/L.

### 2.2. Exclusion Criteria

 Pneumonia defined as
   lung infiltrate on the chest radiograph associated with at lest two of the following conditions: fever, leucocytosis, purulent sputum in which a Gram stain showed one or more types of bacteria.
 Sepsis syndrome defined as
   positive blood cultures and/or fever of >38°C together with positive cultures from suspected sources.
 Renal dialysis
 Glasgow coma scale <8
 Criteria for immediate intubation for cardiopulmonary resuscitation
 Inotropic support with catecolamines at baseline
 Failure of two or more additional organs defined as
   Bilirubin >2 mg/dL, creatinine >2 mg/dL or creatinine clearance <50 mL/min, platelets <100.000/mmc, Hb < 7.5 g/dL.
 Electrocardiogram instability with evidence of life-threatening ventricular arrhythmia.

### 2.3. Patients and Study Design

Ninety five consecutive patients with acute cardiorespiratory insufficiency, admitted from an emergency department to a 16-bed medical ICU/HDU during 2008, were prospectively enrolled in the study. Anthropometric and functional characteristics of the patients are listed in Table 1. 

The preliminary diagnosis was made according to clinical history, physical examination, arterial blood gas, and chest radiography. Subsequent investigations and monitoring took place in the Medical Intensive Care or High Dependency Unit (ICU/HDU) depending on acuity. The following data were collected for all patients. Blood pressure, heart and respiratory rate, Glasgow Coma Scale [11] and APACHE II score [12], arterial blood gases and lactate, full blood count, biochemistry panel, ECG monitoring, and ecocardiographic evaluation.

## 3. Interventions

Gas exchange and lactate blood concentration were assessed at ED arrival and 1, 2, 6, and 24 hours later. Two-hour lactate clearance was calculated as

(1) $$\frac{\left(\text{Lactate}\;\;\text{start}-\text{lactate}\;\;2\;\;\text{hour}\right)}{\text{lactate}\;\;\text{start}\;\;\left(\text{\%}\right)}.$$

Blood gas-analysis and arterial lactate were performed by intermittent blood sampling and co-oximetry (Blood Gas Analyzer, Bayer Health Care Rapid Lab 1265). Inspired fractional O_2_ concentration (FiO_2_) was recorded. 

All patients were treated according to standard practice in our ICU. Protocols for hemodynamic treatment including inotropic support with dobutamine or phosphodiesterase inhibitors, noninvasive positive pressure ventilation (NPPV), light sedation, management of blood glucose, water, and electrolyte derangements were used. No patients in our study were treated with lactate containing solutions, which might have falsely elevated lactate levels. 

The criteria for patients who underwent NPPV were tachypnea with a respiratory rate greater than 25 bpm, pH 7.10–7.35, and PaCO_2_ > 50 mm Hg. Continuous or bi-level positive pressure support were chosen at the discretion of the treating physician and adapted to response of the patient. The criteria to determine when ventilator assistance was discontinued were normal mental status, hemodynamic stability, respiratory rate below 25 bpm, absence of activation of accessory respiratory muscles and paradoxical abdominal motion, arterial pH > 7.33, PaCO_2_ < 70 mmHg, PaO_2_ > 55 mmHg with FIO_2_ > 0.35 without ventilatory support. The patients were discharged from the ICU/HDU when NPPV was switched to oxygen therapy with FIO_2_ < 0.35 for 24 h at least and the patients met the following criteria: cardiorespiratory stability, respiratory rate below 25 bpm, pH > 7.35, PaO_2_ > 55 mmHg, PaCO_2_ < 70 mmHg, absence of fever, leucocytosis, purulent sputum, and metabolic complications. An independent clinical team not involved in the research protocol decided about the discharge.

Primary outcome measures were hospital mortality or endotracheal intubation (negative outcome) and discharge or transfer to a general medical ward (positive outcome).

## 4. Statistical Analysis

The data are expressed as mean and standard deviation for continuous variables, medians, and interquartile range (1st–3rd) for ordinal variables and numbers and frequencies for nominal variables. Normality of data distribution was appraised with Kolmogorov-Smirnov test. Homoscedicity was tested with Levene median test with Lilliefors correction. Based on pilot data and previous works on the prognostic value of lactate in the critically ill [4, 10, 13], the study was powered for an expected sensitivity of 90% with a minimal acceptable confidence limit of 75% [14, 15].

Factors likely to affect outcome were recorded at the outset. These included age >70 years, presence of severe underlying diseases (cancer, pre-existing hepatic, or renal insufficiency) presence of shock, acute renal failure, and baseline laboratory parameters. Factors correlated with negative outcome at *P* < .25 (chi-square or Fisher's exact test, as appropriate) were included in a logistic regression model with the lactate clearance variables. A backward procedure was then used to discard the nonsignificant variables from the analysis. Sensitivity, specificity, and predictive values were calculated according to standard formulae. Receiver operating characteristics curves (ROC) were constructed to evaluate the reliability of 2-hour lactate clearance as prognostic factor. *P* < .05 was considered significant. All statistical analyses were performed with SPSS 15.0 for windows.

## 5. Results

A total of 95 patients were included in the study. All patients had a history of significant underlying pathology, sixty-three had ischemic heart disease (68%), thirty-nine patients had COPD (42%), thirty-five had type 2 diabetes (42%), and twelve had chronic renal failure (8.7%). At enrolment 56 patients were in acute pulmonary oedema (68%), 25 patients had acute respiratory failure for severe exacerbation of COPD (21%), 7 were in cardiogenic shock (6%), and 5 patients presented with acute myocardial infarction. 34 patients required noninvasive positive pressure ventilation (NPPV) (43%). The overall negative outcome rate was 30% (28/95) 12% hospital mortality (11/95). 25 patients required endotracheal intubation and of those 10 (40%) died during their hospital stay. Lactate at baseline was not different between groups but 2-hour lactate and 2-hour lactate clearance were significantly worse in patients with negative outcomes (Table 1). 

The odds ratio for both elevated 2 hour lactate (7.73, *P* = .002) and impaired LACT-2h-clearance (16.11, *P* < .0001) are highly significant for negative outcome but LACT-2h-clearance appears superior. The odds ratios of selected risk factors are displayed in Table 2. 

**Figure 1:**
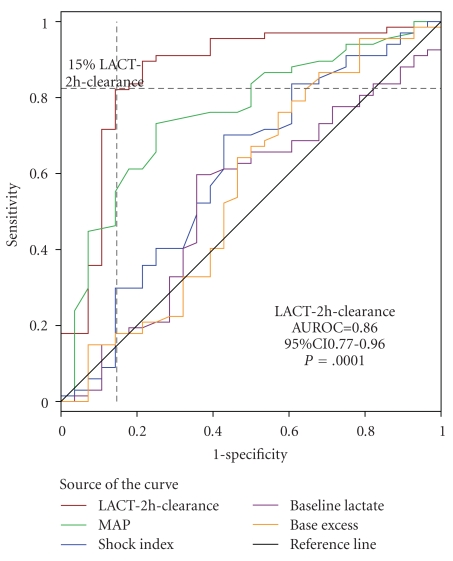
ROC curve for LACT-2h-clearance.

**Figure 2:**
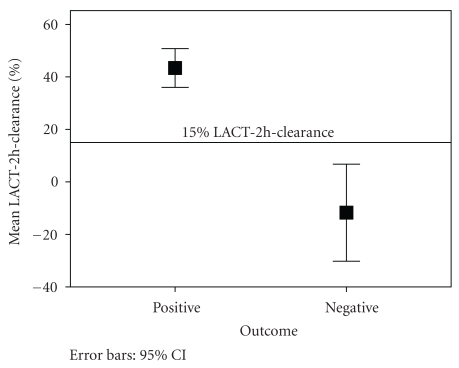
Mean LACT-2h-clearance for positive and negative outcome.

Figure 1 illustrates ROC curves for LACT-2h-clearance. It demonstrates the reliability of LACT-2h-clearance as a predictor of negative outcome, indicating that the best compromise between sensitivity and specificity was obtained for a lactate clearance of 15%. The global reliability of this test to predict mortality is quite good, as confirmed by the value of the area under the ROC (AUROC) curve of 0.86 (*P* ≤ .0001; 95%CI 0.77–0.96) which is comparable to the values previously reported for other risk factors of mortality in similar studies [13].  Figure 2 displays a graphical comparison of mean LACT-2h-clearance between patients with positive and negative outcomes.

When <15% is used as a cut off, LACT-2h-clearance accurately predicted negative outcome with a sensitivity of 86% (95%CI = 67%–95%) and a specificity of 91% (95%CI = 82%–96%). Positive predictive value was 80% (95%CI = 61%–92%) and negative predictive value was 92% (95%CI = 84%–98%). Two-hour lactate clearance also outperforms other markers commonly used in critical care such as baseline lactate (AUROC = 0.46), 2-hour base excess (AUROC = 0.66), shock index (AUROC = 0.61) and MAP (AUROC = 0.75). Two-hour lactate measurements produced AUROC of 0.84 but its sensitivity and specificity were inferior to LACT-2h-clearance given that a cut off of 2.5 mg/dL returned a sensitivity of 82% but a specificity of only 64%. 

Variables identified by the backward logistic regression model as significantly correlated with negative outcome were LACT-2h-clearance less than 15%, 2-hour lactate and MAP less than 90 at presentation. The log likelhood ratio of LACT-2h-clearance less than 15%, was 40.08 (*P* < .001). Other laboratory values, catacolamine use, age, sex, and comorbidites did not predict negative outcome in this model.

## 6. Discussion

The most important result of the present investigation was that LACT-2h-clearance can be feasible and clinically useful as a predictive tool in cardiorespiratory insufficiency. Under the experimental conditions of this study it seems that a cut-off of <15% LACT-2h-clearance is predictive of negative outcome. This measure proved robust even when lactate levels were only mildly elevated at baseline (<3 mmol/L).

Lactate clearance deserves the same diagnostic relevance of other noninvasive markers of O_2_ delivery/consumption/demand mismatch. While tissue pH, O_2_-saturation, PCO_2_, and (prospectively) NADH monitoring could offer a precise “local” picture of cellular dysoxia [16], lactate does not. Nevertheless the systematic checking of 2-hour lactate clearance could be used to tailor the therapy in many cases of cardiac or respiratory insufficiency. It seems to mirror quite precisely hepatic and renal function and perfusion [2, 17] in many critically ill patients. It cannot be substituted by any other single parameter derived from arterial blood gas analysis [18]. Reduced O_2_-delivery is compatible with aerobic respiration if oxygen consumption is simultaneously decreased; this is not an acidifying process “per se” [19]. On the other hand, there are definite “time-windows” in septic syndromes, where the hyper metabolism produces high blood lactate concentrations, in patients who are not yet acidotic. On the contrary, pure acute cardiac or respiratory insufficiencies can be considered acid and lactate producing syndromes, which are mostly associated with reduced acid and lactate removal. Moreover the effects of some therapies (fluid management, inotropes, ventilation, etc.) are not always predictable, due to intrinsic myocardial O_2_-consumption [20] and heart/lung/metabolism interactions. Often even invasive hemodynamic parameters can be puzzling. 

Our patient cohort is made up predominantly of elderly patients with multiple comorbidities, which is representative of an increasingly large segment of the general population and of ICU patients. In such patients invasive ventilation is often better avoided if possible [21]. The decision to include patients with either cardiac and/or respiratory insufficiency was based on the frequent coexistence of these pathologies and the difficulty in precisely differentiating these entities in the emergency department. A robust early prognostic marker, such as LACT-2h-clearance, to identify patients with acute cardiorespiratory insufficiency at high risk of negative outcome irrespective of the ultimate diagnosis is an attractive clinical tool. In keeping with EGDT LACT-2h-clearance may provide a target for clinicians to tailor more aggressive therapy for patients at higher risk of negative outcome. Of equal importance is the identification of patients in whom the current therapy is working and who are likely to have a positive outcome. To our knowledge our work is the first to examine the prognostic value of lactate at 2 hours in this increasingly large patient population. Indeed the cut off of 15% clearance of serum lactate at 2 hours is lower and measured significantly sooner than the bulk of studies investigating the prognostic value of lactate. This is because much of the work in this field has been in relation to septic patients who respond more slowly to therapy than cardiorespiratory patients. 

## 7. Study Limitations

This study has several limitations. First of all, there is a “lag phase” between the initial resolution of the cardiorespiratory crisis and the descending slope of lactate concentration. This is due to a “wash-out” phenomenon linked to flow restoration, plus the O_2_-debt which must be repaid by the liver and kidneys before they can restart the cellular “machinery.” Second, it should be considered that the fast-responder organs are usually pouring their lactate into the blood well before it is detectable on any arterial sample, so that a precise evaluation should be done drawing the effluent blood of each organ (e.g., internal jugular or coronary veins). Third, the lactate/pyruvate (=NADH/NAD) ratio is not routine evaluation, but should be done whenever a hypermetabolic state is suspected [22, 23]. Fourth, there are some drugs and inherited metabolic syndromes which can complicate the picture. In particular inotropic agents are known to increase serum lactate levels. However, there was no correlation in our study between the use of catecholamines and negative outcome as evidenced by the logistic regression analysis.

We do not have data to assess how closely the care team complied with standard protocols and although the same team cared for all patients in the study, there may have been slight variability in the approach by individual clinicians who were in charge of the patients regarding parameters such as inotrope dosage or ventilator settings. These changes may have lead to differences in the quality of care provided, which could introduce a degree of bias into the results. Finally the study included patients with cardiorespiratory failure rather than the patients with a specific pathological diagnosis. Although the results indicate that LACT-2h-clearance predicts outcome irrespective of the underlying pathology the study was not adequately powered to perform subgroup analysis. A larger validation study would be necessary to confirm the predictive values suggested by the current data in specific patient subgroups with purely cardiac or respiratory pathology.

## 8. Conclusions

The systematic monitoring of lactate clearance at 2 hours should be clinically useful in cases of acute cardiac or respiratory insufficiency to identify patients at high risk of negative outcome and, potentially, to increase the intensity of the therapeutic approach. Finally, a 2-hour lactate clearance >15% is highly predictive of positive outcome and may reassure clinicians that the therapeutic approach is appropriate. However, larger, prospective randomized studies will be needed to validate this hypothesis. 

## Figures and Tables

**Table 1:** Patient characteristics.

Variables	Patients (*n* = 95)	Positive outcome (*n* = 67)	Negative outcome (*n* = 28)	*P*
Age (mean ± SD)	76.1 ± 9.4	75.5 ± 9.2	77.8 ± 9.8	NS
M/F	43/52	28/39	5/8	NS
Apache II score	19.4 ± 4.6	19.3 ± 4.2	19.8 ± 5.3	NS

Vital parameters				

Heart rate bpm	114 ± 30.2	117.3 ± 27.8	106.0 ± 34.7	NS
Respiratory rate	34.4 ± 6.8	35.4 ± 6.3	32.3 ± 7.6	NS
MAP mmHg	113.5 ± 32.8	115.6 ± 29.1	94.0 ± 29.3	*P* < .05*

Laboratory values				

WBC, mm^3^	13.3 ± 5.4	13.3 ± 5.3	13.0 ± 5.8	NS
HTC, %	37.8 ± 7.5	38.5 ± 7.2	34.2 ± 8.2	NS
Creatinine, mg/dL	1.37 ± 0.6	1.36 ± 0.75	1.43 ± 0.3	NS
pH	7.25 ± 0.14	7.24 ± 0.08	7.26 ± 0.16	NS
pO_2 _, mmHg	63.4 ± 22.5	67.0 ± 33.1	61.9 ± 16.3	*P* < .05*
pCO_2 _, mmHg	51.4 ± 20.6	51.1 ± 17.6	52.8 ± 26.6	NS
HCO_3 _, mEq/L	21.5 ± 5.8	21.2 ± 5.1	22.5 ± 7.1	NS
Lactate start, mmol/L	4.7 ± 2.6	4.7 ± 2.4	4.7 ± 2.9	NS
Lactate 2 h, mmol/L	3.0 ± 2.0	2.2 ± 0.9	4.7 ± 2.8	*P* < .05*
Lactate clearance at 2 hours(%)	27 ± 43	43 ± 30	−11 ± 47	*P* < .05*

*P < .05, Student *T*-test; NS, Not Significant.

MAP: mean arterial pressure, WBC: white blood count, HTC: hematocrit.

**Table 2:** Relationship between risk factors and negative outcome.

Variables	Positive outcome (*n* = 67)	Negative outcome (*n* = 28)	Odds Ratio (95% CI)	*P*
Age >75	41 (61%)	20 (71%)	1.59 (0.79–3.17)	.34
Male/Female	28/39 (42/58%)	15/13 (54/46%)	N/A	.57
IHD	47 (29%)	16 (31%)	0.57 (0.27–1.16)	.23
Cardiogenic shock	3 (5%)	4 (14%)	3.55 (0.74–16.93)	.07
Myocardial Infarction	4 (6%)	1 (4%)	0.59 (0.06–5.45)	.67
COPD	29 (44%)	10 (38%)	0.73 (0.31–1.69)	.49
Renal Failure	10 (32%)	2 (46%)	0.44 (0.09–2.13)	.31
p0_2_< 60 at 2 hours	7 (17%)	3 (15% )	1.03 (0.25–4.26)	.96
Hemaglobin <10g/dL	5 (7%)	8 (29%)	4.10 (1.21–13.86)	.02
p0_2_< 60 mmHg at presentation	34(38%)	17 (84.6)	1.50 (0.72–3.11)	.005
MAP <90 at presentation	9 (13%)	13(46%)	5.58 (2.01–15.52)	.001
Serum lactate >2.5 mmol/L at 2 hours	23 (37%)	25 (82%)	7.73 (2.60–22.90)	.0002
Lactate clearance <20% at 2 hours	9 (21%)	20 (69.2)	16.11 (6.53–39.70)	<.0001

IHD-ischemic heart disease; COPD–chronic obstructive pulmonary disease; CI-confidence interval.

## Abbreviations

LACT-2h-clearance: (Lactate start – lactate 2 hour)/lactate start (%)
LACT-6h-clearance: (Lactate start – lactate 6 hour)/lactate start (%)
APACHE II: Acute Physiology and Chronic Health Evaluation II
BP: Blood Pressure
BPM: Beats Per Minute
CI: Confidence Interval.
COPD: Chronic Obstructive Pulmonary Disease
ED: Emergency Department
EGDT: Early Goal Directed Therapy
FiO_2_: Inspired Fraction of Oxygen
GCS: Glasgow Coma Score
Hb: Hemoglobin
HDU: High Dependency Unit
Hct: Hematocrit
ICU: Intensive Care Unit
MAP: Mean Arterial Pressure
NPPV: Noninvasive Positive Pressure Ventilation
PaCO_2_: Partial Pressure of Arterial Carbon Dioxide
ROC curve: Receiver Operating Characteristic curve
SpO_2_: Oxygen Saturation
WBC: White Blood Cells
